# Timing of early laparoscopic cholecystectomy for acute calculous cholecystitis revised: Protocol of a systematic review and meta-analysis of results

**DOI:** 10.1186/s13017-019-0285-7

**Published:** 2020-01-03

**Authors:** Giuseppe Borzellino, Safi Khuri, Michele Pisano, Subhi Mansour, Niccolò Allievi, Luca Ansaloni, Yoram Kluger

**Affiliations:** 1 Department of General Surgery, University Hospital of Verona Italy, Piazzale A. STEFANI 1, 37128 Verona, Italy; 2 Department of General Surgery, Rambam Health Care Campus, Haifa, Israel; 3 1st Surgical Unit, Department of Emergency, Papa Giovanni Hospital XXIII, Bergamo, Italy; 4 Department of Emergency and Trauma Surgery, Bufalini Hospital, Cesena, Italy

**Keywords:** Acute cholecystitis, Laparoscopic cholecystectomy, Timing, Morbidity

## Abstract

**Background:**

Early laparoscopic cholecystectomy has been adopted as the treatment of choice for acute cholecystitis due to a shorter hospital length of stay and no increased morbidity when compared to delayed cholecystectomy. However, randomised studies and meta-analysis report a wide array of timings of early cholecystectomy, most of them set at 72 h following admission. Setting early cholecystectomy at 72 h or even later may influence analysis due to a shift towards a more balanced comparison. At this time, the rate of resolving acute cholecystitis and the rate of ongoing acute process because of failed conservative treatment could be not so different when compared to those operated with a delayed timing of 6–12 weeks. As a result, randomised comparison with such timing for early cholecystectomy and meta-analysis including such studies may have missed a possible advantage of an early cholecystectomy performed within 24 h of the admission, when conservative treatment failure has less potential effects on morbidity. This review will explore pooled data focused on randomised studies with a set timing of early cholecystectomy as a maximum of 24 h following admission, with the aim of verifying the hypothesis that cholecystectomy within 24 h may report a lower post-operative complication rate compared to a delayed intervention.

**Methods:**

A systematic review of the literature will identify randomised clinical studies that compared early and delayed cholecystectomy. Pooled data from studies that settled the early intervention within 24 h from admission will be explored and compared in a sub-group analysis with pooled data of studies that settled early intervention as more than 24 h.

**Discussion:**

This paper will not provide evidence strong enough to change the clinical practice, but in case the hypothesis is verified, it will invite to re-consider the timing of early cholecystectomy and might promote future clinical research focusing on an accurate definition of timing for early cholecystectomy for acute cholecystitis.

## Background

Laparoscopic cholecystectomy has been confirmed by the most recent guidelines to be the definitive treatment for acute calculous cholecystitis [1, 2], but the exact timing of the cholecystectomy remains still a matter of debate and aim of studies. Review and meta-analysis have reported clinical trials comparing early to delayed cholecystectomy in which, however, different definitions of early timing for cholecystectomy were adopted. More precisely, cholecystectomy was defined early when performed within 24, 48 or 72 h and even within 96 h following the admission or 1 week following the onset of symptoms [3–10]. Some results of published meta-analysis have been reported to be discordant, but mainly limited to the duration of the intervention [11]. All [2, 5–9] but one [4] found a shorter total hospital stay when cholecystectomy was performed early. Finally, all meta-analysis failed to find any differences in overall morbidity, bile duct injury, and conversion rate when comparing the two sets of timings [2–9].

Performing early cholecystectomy at 72 h or later may be questionable. The study of Arslan, by finding no outcome differences in randomising before and after 72 h during the same admission, invites to not consider 72 h as a break point of the timing of cholecystectomy [12]. Patients operated at 72 h may have had symptoms for 3 to 5 days at the time of cholecystectomy. On a pathological point of view, during this time, lymphoplasmacellular infiltration replaces polymorphonuclear cells, inducing a diagnostic shift from acute to chronic cholecystitis [13, 14]. Moreover, in the study of Gutt et al, discharges were programmed as soon as possible following a 72-h period of medical treatment in the patients randomised in the delayed group [15], and in the trial of Johansson et al., mean duration of symptoms in the delayed group was reported to be 58 h [16]. This could be an advantage in setting the early timing at 72 h but it may have influenced analysis by a shift towards more balanced comparisons; patients submitted to early cholecystectomy at 72 h might not eventually be at a much higher risk of complications compared to those operated later during a second hospitalisation. On the other hand, medical treatment has a risk of failure with cumulative rates of 0 to 30% reported in the delayed groups [15–23] without any data provided for patients in the early groups. It can be therefore hypothesised that even in the early groups a rate of acute cholecystitis may be resolving and a rate may not be improving or worsening at the time of surgery, as it happened in the delayed group. As a result, the later early cholecystectomy is performed, the more patients within early and delayed cholecystectomy may have an overlaying risk of complications.

Until now and to the best of our knowledge no meta-analysis has reported results focused on immediate versus delayed cholecystectomy. By including different timing of early cholecystectomy, the conducted meta-analysis may have therefore missed the potential benefit, in terms of complications, of early cholecystectomy performed within 24 h following the admission.

## Objective

The study will be performed to explore such a hypothesis by performing a systematic review of the literature and a meta-analysis of randomised clinical trials that compared early cholecystectomy performed within 24 h from the admission and delayed cholecystectomy in patients with an acute cholecystitis fit for an urgent surgical approach.

## Methods

The meta-analysis will be performed according to the PRISMA statement for reporting reviews and meta-analysis [24]. The present protocol has been submitted for publication on the PROSPERO database for meta-analysis.

## Literature search to identify included studies

A search of studies will be conducted on PubMed by two independent Authors. A term strategy based on PICOS acronym will be adopted, using subject headings and text words that allow to identify randomised studies including patients with acute cholecystitis, submitted to laparoscopic cholecystectomy, performed early after admission or delayed and reported overall complication rate. No limits of dates nor limits of language will be imposed. The following used search strategy will be used for PubMed:

Search: ((((((((cholecystitis[MeSH Terms]) OR acute cholecystitis[MeSH Terms]) OR cholecystitis, acute[MeSH Terms])) AND ((((laparoscop*) OR celioscop*) OR coelioscop*) OR peritoneoscop*)) AND ((cholecystectomy) OR cholecystectomies)) AND (((((immediate) OR early) OR urgent) OR delayed) OR timing)) AND (((morbidit*) OR complication*) OR post-operative)) AND random*

Literature search will be completed by consulting the Cochrane Library, Embase and Clinical.trial.gov, and hand searching by reviewing all the references of the articles found to be of interest for this paper, including reviews and meta-analysis. Unpublished studies or data from presentations to congress were not considered.

## Study selection

Two authors will independently assess studies. A first selection will be made based on the title and abstract. Papers will be selected for a full-text reading only if it is reported the study compares two different timing of laparoscopic cholecystectomy for patients with acute cholecystitis. Papers will not be selected if the title or abstract report clearly that the study is not comparative or that some categories of patients such as elderly will be excluded.

A second selection will be performed based on the full-text reading; papers will be included in the review only if it is specified the study is a comparative randomised trial, two different timing of laparoscopic cholecystectomy are compared, criteria for the diagnosis of acute cholecystitis are clearly defined, as well as population study and the different timing of surgery, and finally only if data on post-operative complications are reported, together with eventually bile duct injury and/or conversion rates and/or mortality.

Studies will be excluded in case they do not define early cholecystectomy by an exact numerical timing of the intervention but by imprecise indications such as immediate or as soon as possible, in case they do not define the population study, exclude some categories of patients and include patients with a different disease than acute cholecystitis.

## Data collection

Data will be collected by two authors independently and reported in a pre-prepared sheet. Only ITT data will be collected. The primary outcome of the study will be the post-operative complication rates. This is the main component that may be directly influenced by the timing of cholecystectomy and that could change the course of the story of patients. Other parameters such as operative time, and intra-operative complications not requiring conversion could be used to assess the role of timing but they may be more subject to the influence of third factors and they may not finally change the course of the treatment. Furthermore, three other secondary outcomes will be registered; it is about bile duct injury, conversion and mortality, considered of interest since they all may change the course of the treatment:

In case of missing or not clear data on post-operative complications, authors will be contacted as an attempt to obtain or clarify the relevant data. Disagreements will be managed by discussing and asking a third author to decide for the final decision.

## Quality assessment of the selected studies

Risk of bias will be independently assessed by two authors using the Cochrane collaborations tool for assessing risk of bias. Quality assessment will therefore focus on risk of bias arising from the randomisation process in the included studies, the allocation concealment, blinding, missing outcome data, measurement of the outcome and selective reporting.

Three different levels of risk, which are low, uncertain or high, will be incorporated according to the findings of the “risk of bias” assessment.

Publication bias will be explored by using a funnel plot in the presence of at least 10 trials [25, 26] and plan to use asymmetry of trial size against treatment effect to assess this bias.

## Statistical methods

Since primary and secondary outcomes are dichotomous variables, relative risk and its 95% confidence interval will be calculated. Since studies will be selected based on defined criteria: all will be randomised clinical trials, including patients with well-defined acute cholecystitis, submitted to early or delayed cholecystectomy, no clinical nor methodological heterogeneity could be expected. We will therefore use a fixed-effect model [27] for the pooling RR and its 95% confidence interval.

Heterogeneity will be estimated with the χ^2^ test and the *I*^2^ statistic. Heterogeneity will be excluded when *I*^2^ will be less than 25% and considered moderate when less than 50% [28, 29]. The meta-analysis will be conducted using the ReviewManager (RevMan) computer programme Version 5.3 [30].

## Interpretation of findings

The hypothesis that immediate laparoscopic cholecystectomy may reduce post-operative complications will be explored by pooling data of studies that settled the early intervention within 24 h after the admission and assessing whether immediate intervention reduce the rate of post-operative complication compared to delayed cholecystectomy.

On a methodological point of view, the only difference among selected studies is the different timing of early cholecystectomy. This allows to compare the results of the first group of studies that settled early cholecystectomy within 24 h from the admission to the results of a second group of included studies that settled early cholecystectomy later during the same admission. Such a comparison will complete the analysis of the results on post-operative morbidity providing furthermore a statistical evaluation of the results.

In case of heterogeneity, an analysis based on the number of included patients or based on the year of publication will also be performed. Studies including a limited number of patients are at risk of a II type error. By having calculated that 200 patients per arm would be required to consider a statistically significant 10% decrease from 20 to 10% of complications after respectively delayed and early cholecystectomy with alpha 0.05 and beta 0.8, a sub-group analysis will be performed by analysing studies that have included less or more than 200 patients in at least one arm. The first randomised trial having been published more than 20 years ago and was related to an older experience, while more recent publications have less than a couple years, the role of gained experience will be assessed as a potential for heterogeneity by sub-group analysis comparing studies published more than 10 years ago and studies published less than 10 years ago.

A chi-square test for sub-group differences will be performed, setting a *p* value at 0.05 to identify any significant differences.

## Sensitivity analysis

A sensitivity analysis will be performed by comparing the fixed-effect model [27] and the random effect model using the DerSimonian Laird method [31]. The study is designed to explore immediate cholecystectomy within 24 h after the admission, but the theoretical key point of early cholecystectomy is potential waiting until 72 h; we planned a sensitivity analysis by including all the studies setting early intervention at less than 72 h in the immediate early cholecystectomy group in comparison with a delayed group. We will complete the sensitivity analysis by evaluating the role of excluded studies because of incomplete information about the exact timing or methodological aspect of the studies including each of them in the immediate or delayed group according to the indicated or supposed timing of cholecystectomy.

## Considering the quality of evidence

The quality of evidence will be evaluated according to the GRADE recommendations, the Cochrane Library [32]. Five domains that can lower the certainty of a body of evidence will therefore be considered as follows: risk of bias and inconsistency across studies; indirectness of studies that do not directly answer or apply to the review question; imprecision of studies reporting few people or events or wide confidence intervals allowing different conclusions and publication bias.

Rating up of the evidence will be considered in case of large effect.

## Discussion

The study aims to evaluate the role of laparoscopic cholecystectomy performed within 24 h from the admission on post-operative complications rates in patients with acute cholecystitis. A 24-h mark for surgery has never been investigated by meta-analysis. The aim is based on the hypothesis that published meta-analysis, by including RCTs that compared early cholecystectomy up to 96 h from admission and even 1 week from symptoms with delayed cholecystectomy at 6–12 weeks may have missed the potential of urgent cholecystectomy performed within 24 h from the admission. To the best of our knowledge, no RCT comparing laparoscopic cholecystectomy within 24 and 72 or 96 h has been published until now. We proposed therefore a meta-analysis of results of RCTs comparing early and delayed cholecystectomy but including only those that considered early timing at no later than 24 h from admission. A sub-group analysis will be also performed in order to assess whether such a comparison will show a difference other meta-analysis have not shown until now. The present meta-analysis is not the more appropriate methodological approach to evaluate the efficacy of such a treatment strategy. However, in case it confirms our hypothesis that cholecystectomy within 24 h may reduce post-operative complication compared to delayed cholecystectomy while early cholecystectomy set at later timing did not, it could put forward a revision of the timing of early cholecystectomy for acute cholecystitis and provide the basis for future clinical research.

## Review status

Searching relevant studies in the databases will begin in September. The review is expected to be complete by November 2019.

## Data Availability

The datasets used and/or analysed during the current study will be available from the corresponding author on reasonable request.
